# Supplementary material for: An accurate and interpretable model for antimicrobial resistance in pathogenic Escherichia coli from livestock and companion animal species
Source: PLoS One. 2023 Aug 24;18(8):e0290473. doi: 10.1371/journal.pone.0290473 (PMC10449230; doi:10.1371/journal.pone.0290473)
Supplement: S1 File — (PDF) [file pone.0290473.s001.pdf]

Supplemental Information for "An accurate and interpretable model for antimicrobial resistance in pathogenic *Escherichia coli* from livestock and companion animal species"

S1. Resistance phenotypes by antibiotic and breakpoint. Listed are number of samples with each resistance phenotype separated by reference breakpoint and antibiotic. Resistance phenotypes are; I = intermediate, NI = non-interpretable, R = resistant, and S = sensitive.

BP	Antibiotic	I	NI	R	S
CLSI	amikacin	15	1	7	319
	ampicillin		27	571	46
	cefazolin	18	109	91	124
	cefovecin	2		26	105
	cefpodoxime	2		54	138
	ceftazidime	8		26	93
	ceftiofur			51	146
	cephalexin	29	18	97	49
	co-amoxiclav$_a$	1	30	231	104
	doxycycline*		93	52	3
	enrofloxacin	7	124	69	410
	gentamicin	5	1	48	288
	marbofloxacin	2		41	256
	minocycline*		2	21	
	orbifloxacin	8		42	248
	pradofloxacin	1		41	257
	TZP$_c$	4		9	180
ECOFF	amikacin*				175
	amoxicillin			57	106
	ampicillin			91	82
	azithromycin*		34		116
	cefazolin			26	149
	cefovecin		233		
	cefpodoxime			19	153
	ceftazidime		212	49	128
	ceftiofur		2	75	340
	cephalexin*		20		152
	cephalothin*				1
	chloramphenicol		3	53	461
	chlortetracycline		183		
	clarithromycin		151		
	clindamycin		464		
	danofloxacin		300		
	doxycycline			69	300
	enrofloxacin		30	94	246
	erythromycin		315		

florfenicol*	98		365
gamithromycin	146		
gentamicin	17	111	510
imipenem*	511	4	2
marbofloxacin	67		
minocycline*			1
neomycin		126	337
nitrofurantoin*			1
novobiocin	163		
orbifloxacin	67		
oxacillin	152		
oxytetracycline	346		
penicillin	615		
pradofloxacin	67		
rifampin	152		
spectinomycin	28	100	335
streptomycin		44	119
sulphadimethoxine	463		
sulphathiazole	163		
tetracycline	2	270	554
tiamulin	300		
ticarcillin	146		
ticarcillin clavulanic acid	4	21	121
tildipirosin	146		
tilmicosin	300		
TMP/SMX$_b$	189	205	552
tulathromycin	271		
tylosin tartrate	434		
TZP$_c$*		5	167
vancomycin	1		

[a] amoxicillin-clavulanic acid [b] trimethoprim-sulphamethoxazole [c] piperacillin-tazobactam
* Excluded from analysis due to low sample size.

S2. Antibiotic and Host Animals without ECOFF or CLSI breakpoints. Listed are the number of antibiotic and host animal combinations that did not have interpretable phenotypes under CLSI or ECOFF breakpoints at the time of writing.

**Antibiotic**	**Host Animal**	**n**
cefovecin	cat, dog	235
chlortetracycline	cattle, swine	183
clarithromycin	horse	151
clindamycin	cattle, swine, chicken, turkey, dog	465
danofloxacin	cattle, swine	300
enrofloxacin	swine	20
erythromycin	chicken, turkey, horse, dog	316
gamithromycin	cattle, swine	146
marbofloxacin	cat	67
novobiocin	chicken, turkey	164
orbifloxacin	cat	67
oxacillin	horse, dog	152
oxytetracycline	cattle, swine, chicken, turkey	347
penicillin	cattle, swine, chicken, turkey, horse, dog	616
pradofloxacin	cat	67
rifampin	horse, dog	152
sulphadimethoxine	cattle, swine, chicken, turkey	464
sulphathiazole	chicken, turkey	164
tiamulin	cattle, swine	300
ticarcillin	horse	146
tildipirosin	cattle, swine	146
tilmicosin	cattle, swine	300
tulathromycin	cattle, swine	271
tylosin tartrate	cattle, swine, chicken, turkey	435
vancomycin	dog	1

S3. Predictor importance by type. Listed are the summed importance of Gene&Binary&Count predictors by type. “Animal All” refers to host animal main effects with any interaction terms. “Gene All” refers to main gene, binary, and count predictors.

**BP**	**Antibiotic**	**Gene Main**	**Animal Main**	**Binary**	**Count**	**Interactions**	**Animal All**	**Gene All**
CLSI	amikacin	16		1.2	20	0.37	0.37	37
	co-amoxiclav$_a$	11		1.9	11			23
	ampicillin	28	0.076	3.7	46	3.7	3.8	78
	cefazolin	120	0.52	0.48	120	0.012	0.53	230
	cefovecin	27	0.34	2.9	20	0.69	1	50
	cefpodoxime	74		3.2	35			110
	ceftazidime	9.6		1.3	5.7			17
	ceftiofur	91		3.3	42			140
	cephalexin	50		2	30			82
	enrofloxacin				0.48			0.48
	gentamicin	4.8			27	0.54	0.54	32
	marbofloxacin				0.26			0.26
	orbifloxacin				0.44			0.44
	TZP$_c$	140		4.7	27			170
	pradofloxacin				0.26			0.26
ECOFF	amoxicillin	47		3.3	24			75
	ampicillin				0.96			0.96
	cefazolin	17	0.0054	2.4	21	2.2	2.2	40
	cefpodoxime	73		3	90			170
	ceftazidime	9.8	0.069		15	2.5	2.5	25
	ceftiofur	65		1.2	34			100
	chloramphenicol	13	6.1	0.49	19	0.14	6.3	32
	doxycycline	8.9		0.82	25			35
	enrofloxacin				0.39			0.39
	gentamicin	46	0.092		54	0.25	0.34	100
	neomycin	26	0.09		30	1.1	1.2	55
	spectinomycin	25	0.13		32	0.73	0.86	57
	streptomycin	35	0.14	0.54	51	0.033	0.18	87
	tetracycline	35	0.43	2.3	30	2.4	2.8	67
	co-ticarclav	31		1.8	34			67
	TMP/SMX$_b$	6.9	0.74	2.3	20	12	13	29

[a] amoxicillin-clavulanic acid [b] trimethoprim-sulphamethoxazole [c] piperacillin-tazobactam

S4. Important predictors in fitted *Gene&Binary&Count* elastic net model by antibiotic. Listed are the important predictors identified by Gene&Binary&Count models for each antibiotic are listed. Predictor importance is estimated by the absolute value of the predictor coefficient in the model. Animal host interaction terms are not shown.

BP	Antibiotic	Important Predictors
CLSI	amikacin	AAC(3)-VIa, AAC(6')-Ib-cr, AAC(6')-Ib-cr5, AAC(6')-Ib', AAC(6')-Ib4, AAC(6')-IIc, binary, count
	co-amoxiclav$_a$	binary, blaEC, blaTEM-1, blaTEM-1B, blaTEM-1C, blaTEM-235, count, CTX-M-14
	ampicillin	binary, blaEC, blaEC-13, blaEC-18, blaEC-19, blaEC-5, blaEC-8, blaTEM-1, blaTEM-1B, CMY-2, count, dog
	cefazolin	binary, blaEC, blaEC-13, blaEC-18, blaEC-19, blaEC-5, blaEC-8, blaTEM-1, blaTEM-150, blaTEM-1A, blaTEM-1B, CMY-2, count, dog, horse
	cefovecin	binary, blaEC, blaEC-13, blaEC-18, blaEC-19, blaEC-5, blaEC-8, blaTEM-1, blaTEM-104, blaTEM-105, blaTEM-150, blaTEM-1A, blaTEM-1B, blaTEM-1C, blaTEM-235, cat, CMY-2, count, CTX-M-14, dog
	cefpodoxime	binary, blaEC, blaEC-13, blaEC-18, blaEC-19, blaEC-5, blaEC-8, blaTEM-1, blaTEM-102, blaTEM-104, blaTEM-150, blaTEM-1A, blaTEM-1B, CMY-2, count, CTX-M-116
	ceftazidime	binary, blaEC-15, blaEC-5, blaTEM-102, blaTEM-104, CMY-2, count, CTX-M-15
	ceftiofur	binary, blaEC, blaEC-13, blaEC-15, blaEC-18, blaEC-5, blaEC-8, blaTEM, blaTEM-1, blaTEM-102, blaTEM-105, blaTEM-150, blaTEM-1A, blaTEM-1C, blaTEM-235, CMY-2, count, CTX-M-15, CTX-M-55, OXA-1, OXA-395, ROB-1
	cephalexin	binary, blaEC, blaEC-13, blaEC-19, blaEC-5, blaEC-8, blaTEM, blaTEM-1, blaTEM-104, blaTEM-105, blaTEM-150, blaTEM-1A, blaTEM-1B, CMY-2, count
	enrofloxacin	count
	gentamicin	AAC(3)-Iid, AAC(3)-IId, AAC(3)-VIa, AAC(6')-Ib', AAC(6')-Ib4, ANT(2")-Ia, count
	marbofloxacin	count
	orbifloxacin	count
	TZP$_c$	binary, blaEC, blaEC-13, blaEC-15, blaEC-18, blaEC-19, blaEC-5, blaEC-8, blaTEM-1, blaTEM-102, blaTEM-150, blaTEM-1A, blaTEM-1B, CARB-2, CMY-107, CMY-130, CMY-2, count, CTX-M-116, CTX-M-14, CTX-M-15, CTX-M-27, CTX-M-55, OXA-1, ROB, ROB-1, ROB-2
	pradofloxacin	count
ECOFF	amoxicillin	binary, blaEC, blaEC-13, blaEC-15, blaEC-18, blaEC-19, blaEC-5, blaEC-8, blaTEM-1, blaTEM-1B, chicken, CMY-2, count, OXA-60, OXA-60b, turkey
	ampicillin	binary, blaEC, blaEC-13, blaEC-15, blaEC-18, blaEC-19, blaTEM-1, blaTEM-102, blaTEM-141, CMY-2, count, CTX-M-1
	cefazolin	binary, blaEC, blaEC-19, blaEC-8, blaTEM-1, blaTEM-104, blaTEM-150, blaTEM-1A, blaTEM-1B, blaTEM-34, cat, CMY-2, count, horse, OXA-364, OXA-787
	cefpodoxime	binary, blaEC, blaEC-18, blaEC-19, blaEC-5, blaEC-8, blaTEM-1, blaTEM-1C, blaTEM-235, CMY-2, count, CTX-M-14, CTX-M-15, OXA-364, OXA-787
	ceftazidime	blaTEM-1, blaTEM-150, blaTEM-1A, CMY-2, count, CTX-M-1, CTX-M-15, CTX-M-55, horse, SHV-12
	ceftiofur	binary, blaA_Mtub, blaEC, blaEC-13, blaEC-15, blaEC-18, blaEC-19, blaEC-5, blaR1, blaTEM-1, blaTEM-102, blaTEM-12, blaTEM-141, blaTEM-150, blaTEM-1A, blaTEM-1B, blaTEM-1C, blaTEM-1D, blaTEM-235, blaZ, blaZ01, blaZ8, CARB-2, chicken, CMY-2, count, CTX-M-1, CTX-M-55, HER-3, HERA-3, horse, OXA-60, OXA-60b, PDC-10, PDC-113, SHV-12
	chloramphenicol	binary, catA1, catB3, cmlA, count, floR, oqxB9
	doxycycline	binary, count, tet(A), tet(B), tet34
	enrofloxacin	count
	gentamicin	AAC(3)-Ib, AAC(3)-II, AAC(3)-Iid, AAC(3)-IId, AAC(3)-IIe, AAC(3)-IIg, AAC(3)-VIa, AAC(6')-Ib4, cat, count, swine

neomycin	AAC(3)-IV, AAC(3)-IVa, AAC(3)-VIa, APH(3')-Ia, APH(3')-IIa, APH(3')-IIb, binary, chicken, count
spectinomycin	aadA1, aadA10, aadA12, aadA2, aadA5, ANT(3")-Ia, ant(3")-Ih/aac(6')-IId, ANT(3")-Ii-AAC(6')-IId, cattle, count, swine, turkey
streptomycin	AAC(3)-VIa, aadA1, aadA2, ANT(3")-Ia, APH(3")-Ib, APH(6)-Id, binary, chicken, count, turkey
tetracycline	binary, cattle, chicken, count, dog, otr(A), tet(A), tet(B), tet(C), tet(M), tet34, turkey
co-ticarclav	binary, blaEC-13, blaEC-19, blaI, blaTEM-1, blaTEM-105, blaTEM-1B, CMY-2, count, CTX-M-1, CTX-M-15, CTX-M-55
TMP/SMX$_b$	binary, cat, cattle, chicken, count, dfrA1, dfrA12, dfrA17, dfrA5, horse, swine, turkey

[a] amoxicillin-clavulanic acid [b] trimethoprim-sulphamethoxazole [c] piperacillin-tazobactam

S5. Evaluation metrics for all models by antibiotic and breakpoint. Listed are the performance metrics for all models on the test set. Values are separated by breakpoint, antibiotic, and model.

BP	Antibiotic	Model	Specificity	Sensitivity	PPV	NPV	Accuracy	F1
CLSI	amikacin	Gene	1	0.6	1	0.069	0.61	0.75
CLSI	co-amoxiclav	Gene	0.38	0.81	0.37	0.82	0.51	0.51
CLSI	ampicillin	Gene	0.8	0.92	0.33	0.99	0.81	0.49
CLSI	cefazolin	Gene	0.77	0.96	0.83	0.94	0.87	0.89
CLSI	cefovecin	Gene	1	0.9	1	0.75	0.93	0.95
CLSI	cefpodoxime	Gene	0.83	1	0.93	1	0.95	0.97
CLSI	ceftazidime	Gene	1	0.95	1	0.87	0.96	0.97
CLSI	cephalexin	Gene	0.5	0.7	0.35	0.81	0.56	0.47
CLSI	enrofloxacin	Gene	0.88	0.59	0.96	0.29	0.63	0.73
CLSI	gentamicin	Gene	0.82	1	0.97	1	0.97	0.98
CLSI	marbofloxacin	Gene	1	0.31	1	0.2	0.41	0.47
CLSI	orbifloxacin	Gene	1	0.46	1	0.27	0.55	0.63
CLSI	TZP	Gene	1	0.82	1	0.13	0.82	0.9
CLSI	pradofloxacin	Gene	0.89	0.33	0.94	0.19	0.41	0.49
CLSI	ceftiofur	Gene	0.82	0.97	0.94	0.9	0.93	0.95
ECOFF	ampicillin	Gene	0.79	0.94	0.8	0.94	0.86	0.86
ECOFF	cefazolin	Gene	0.83	0.67	0.95	0.33	0.69	0.78
ECOFF	cefpodoxime	Gene	0.5	0.97	0.94	0.67	0.91	0.95
ECOFF	ceftazidime	Gene	0.8	1	0.93	1	0.94	0.96
ECOFF	chloramphenicol	Gene	0.91	0.96	0.99	0.71	0.95	0.97
ECOFF	doxycycline	Gene	0.86	0.97	0.97	0.86	0.95	0.97
ECOFF	enrofloxacin	Gene	0.42	0.88	0.8	0.57	0.75	0.84
ECOFF	gentamicin	Gene	0.96	0.96	0.99	0.85	0.96	0.98
ECOFF	tetracycline	Gene	0.94	0.96	0.97	0.93	0.96	0.97
ECOFF	TMP/SMX	Gene	0.93	1	0.97	1	0.98	0.99
ECOFF	amoxicillin	Gene	0.92	0.95	0.95	0.92	0.94	0.95
ECOFF	ceftiofur	Gene	0.87	0.97	0.97	0.87	0.95	0.97
ECOFF	neomycin	Gene	0.77	1	0.92	1	0.94	0.96
ECOFF	spectinomycin	Gene	0.95	0.94	0.98	0.83	0.94	0.96
ECOFF	streptomycin	Gene	0.89	0.92	0.96	0.8	0.91	0.94
ECOFF	co-ticarclav	Gene	0.4	0.88	0.88	0.4	0.8	0.88
CLSI	amikacin	Gene&Binary	1	0.6	1	0.069	0.61	0.75
CLSI	co-amoxiclav	Gene&Binary	0.64	0.81	0.5	0.88	0.69	0.62
CLSI	ampicillin	Gene&Binary	0.91	0.75	0.47	0.97	0.9	0.58
CLSI	cefazolin	Gene&Binary	0.82	0.96	0.86	0.95	0.89	0.91
CLSI	cefovecin	Gene&Binary	0.83	0.95	0.95	0.83	0.93	0.95
CLSI	cefpodoxime	Gene&Binary	0.67	1	0.88	1	0.9	0.93
CLSI	ceftazidime	Gene&Binary	1	0.95	1	0.87	0.96	0.97
CLSI	cephalexin	Gene&Binary	0.73	0.8	0.53	0.9	0.75	0.64
CLSI	enrofloxacin	Gene&Binary	0.88	0.59	0.96	0.29	0.63	0.73
CLSI	gentamicin	Gene&Binary	0.82	1	0.97	1	0.97	0.98
CLSI	marbofloxacin	Gene&Binary	1	0.31	1	0.2	0.41	0.47
CLSI	orbifloxacin	Gene&Binary	1	0.46	1	0.27	0.55	0.63
CLSI	TZP	Gene&Binary	1	0.82	1	0.13	0.82	0.9
CLSI	pradofloxacin	Gene&Binary	0.89	0.33	0.94	0.19	0.41	0.49
CLSI	ceftiofur	Gene&Binary	0.82	0.97	0.94	0.9	0.93	0.95
ECOFF	ampicillin	Gene&Binary	0.79	1	0.81	1	0.89	0.89
ECOFF	cefazolin	Gene&Binary	0.83	0.6	0.95	0.29	0.64	0.73
ECOFF	cefpodoxime	Gene&Binary	0.5	1	0.94	1	0.94	0.97
ECOFF	ceftazidime	Gene&Binary	0.8	1	0.93	1	0.94	0.96
ECOFF	chloramphenicol	Gene&Binary	0.91	1	0.99	1	0.99	0.99
ECOFF	doxycycline	Gene&Binary	0.86	0.98	0.97	0.92	0.96	0.98
ECOFF	enrofloxacin	Gene&Binary	0.16	1	0.76	1	0.77	0.86
ECOFF	gentamicin	Gene&Binary	0.96	0.99	0.99	0.96	0.98	0.99
ECOFF	tetracycline	Gene&Binary	0.98	0.98	0.99	0.96	0.98	0.99
ECOFF	TMP/SMX	Gene&Binary	0.95	1	0.98	1	0.99	0.99
ECOFF	amoxicillin	Gene&Binary	0.92	0.95	0.95	0.92	0.94	0.95
ECOFF	ceftiofur	Gene&Binary	0.87	0.97	0.97	0.87	0.95	0.97
ECOFF	neomycin	Gene&Binary	0.81	1	0.93	1	0.95	0.96
ECOFF	spectinomycin	Gene&Binary	1	0.87	1	0.69	0.9	0.93
ECOFF	streptomycin	Gene&Binary	0.89	0.96	0.96	0.89	0.94	0.96
ECOFF	co-ticarclav	Gene&Binary	0.4	0.88	0.88	0.4	0.8	0.88
CLSI	amikacin	Gene&Count	1	0.97	1	0.5	0.97	0.98
CLSI	co-amoxiclav	Gene&Count	1	1	1	1	1	1
CLSI	ampicillin	Gene&Count	1	0.92	1	0.99	0.99	0.96
CLSI	cefazolin	Gene&Count	0.95	1	0.96	1	0.98	0.98
CLSI	cefovecin	Gene&Count	1	0.95	1	0.86	0.96	0.98
CLSI	cefpodoxime	Gene&Count	1	0.93	1	0.86	0.95	0.96
CLSI	ceftazidime	Gene&Count	1	0.95	1	0.87	0.96	0.97
CLSI	cephalexin	Gene&Count	1	1	1	1	1	1
CLSI	enrofloxacin	Gene&Count	1	1	1	1	1	1
CLSI	gentamicin	Gene&Count	0.91	1	0.98	1	0.99	0.99
CLSI	marbofloxacin	Gene&Count	1	1	1	1	1	1
CLSI	orbifloxacin	Gene&Count	1	1	1	1	1	1
CLSI	TZP	Gene&Count	1	0.97	1	0.5	0.97	0.99
CLSI	pradofloxacin	Gene&Count	1	0.98	1	0.9	0.98	0.99
CLSI	ceftiofur	Gene&Count	1	0.97	1	0.92	0.98	0.98
ECOFF	ampicillin	Gene&Count	0.79	1	0.81	1	0.89	0.89
ECOFF	cefazolin	Gene&Count	1	0.9	1	0.67	0.92	0.95
ECOFF	cefpodoxime	Gene&Count	1	0.97	1	0.8	0.97	0.98
ECOFF	ceftazidime	Gene&Count	0.9	1	0.96	1	0.97	0.98
ECOFF	chloramphenicol	Gene&Count	1	0.96	1	0.73	0.96	0.98
ECOFF	doxycycline	Gene&Count	1	0.98	1	0.93	0.99	0.99
ECOFF	enrofloxacin	Gene&Count	1	0.98	1	0.95	0.99	0.99
ECOFF	gentamicin	Gene&Count	1	1	1	1	1	1
ECOFF	tetracycline	Gene&Count	1	1	1	1	1	1
ECOFF	TMP/SMX	Gene&Count	1	1	1	1	1	1
ECOFF	amoxicillin	Gene&Count	1	0.95	1	0.92	0.97	0.98
ECOFF	ceftiofur	Gene&Count	1	0.99	1	0.94	0.99	0.99
ECOFF	neomycin	Gene&Count	1	1	1	1	1	1

ECOFF	spectinomycin	Gene&Count	1	0.99	1	0.95	0.99	0.99
ECOFF	streptomycin	Gene&Count	1	1	1	1	1	1
ECOFF	co-ticarclav	Gene&Count	0.6	0.96	0.92	0.75	0.9	0.94
CLSI	amikacin	Gene&Binary&Count	1	0.97	1	0.5	0.97	0.98
CLSI	co-amoxiclav	Gene&Binary&Count	1	1	1	1	1	1
CLSI	ampicillin	Gene&Binary&Count	1	0.92	1	0.99	0.99	0.96
CLSI	cefazolin	Gene&Binary&Count	0.95	1	0.96	1	0.98	0.98
CLSI	cefovecin	Gene&Binary&Count	1	1	1	1	1	1
CLSI	cefpodoxime	Gene&Binary&Count	1	0.93	1	0.86	0.95	0.96
CLSI	ceftazidime	Gene&Binary&Count	1	0.95	1	0.87	0.96	0.97
CLSI	cephalexin	Gene&Binary&Count	1	1	1	1	1	1
CLSI	enrofloxacin	Gene&Binary&Count	1	1	1	1	1	1
CLSI	gentamicin	Gene&Binary&Count	0.91	1	0.98	1	0.99	0.99
CLSI	marbofloxacin	Gene&Binary&Count	1	1	1	1	1	1
CLSI	orbifloxacin	Gene&Binary&Count	1	1	1	1	1	1
CLSI	TZP	Gene&Binary&Count	1	0.95	1	0.33	0.95	0.97
CLSI	pradofloxacin	Gene&Binary&Count	1	0.98	1	0.9	0.98	0.99
CLSI	ceftiofur	Gene&Binary&Count	1	0.97	1	0.92	0.98	0.98
ECOFF	ampicillin	Gene&Binary&Count	0.79	1	0.81	1	0.89	0.89
ECOFF	cefazolin	Gene&Binary&Count	1	0.9	1	0.67	0.92	0.95
ECOFF	cefpodoxime	Gene&Binary&Count	1	0.97	1	0.8	0.97	0.98
ECOFF	ceftazidime	Gene&Binary&Count	0.9	1	0.96	1	0.97	0.98
ECOFF	chloramphenicol	Gene&Binary&Count	1	0.96	1	0.73	0.96	0.98
ECOFF	doxycycline	Gene&Binary&Count	1	0.98	1	0.93	0.99	0.99
ECOFF	enrofloxacin	Gene&Binary&Count	1	0.98	1	0.95	0.99	0.99
ECOFF	gentamicin	Gene&Binary&Count	1	1	1	1	1	1
ECOFF	tetracycline	Gene&Binary&Count	1	1	1	1	1	1
ECOFF	TMP/SMX	Gene&Binary&Count	1	1	1	1	1	1
ECOFF	amoxicillin	Gene&Binary&Count	1	0.95	1	0.92	0.97	0.98
ECOFF	ceftiofur	Gene&Binary&Count	1	0.99	1	0.94	0.99	0.99
ECOFF	neomycin	Gene&Binary&Count	1	1	1	1	1	1
ECOFF	spectinomycin	Gene&Binary&Count	1	0.97	1	0.91	0.98	0.98
ECOFF	streptomycin	Gene&Binary&Count	1	1	1	1	1	1
ECOFF	co-ticarclav	Gene&Binary&Count	0.8	0.96	0.96	0.8	0.93	0.96
CLSI	amikacin	Full	1	0.78	1	0.12	0.78	0.87
CLSI	co-amoxiclav	Full	0.28	0.81	0.33	0.76	0.44	0.47
CLSI	ampicillin	Full	0.79	0.92	0.32	0.99	0.81	0.48
CLSI	cefazolin	Full	0.77	0.88	0.81	0.85	0.83	0.85
CLSI	cefovecin	Full	1	1	1	1	1	1
CLSI	cefpodoxime	Full	0.83	1	0.93	1	0.95	0.97
CLSI	ceftazidime	Full	1	0.95	1	0.87	0.96	0.97
CLSI	cephalexin	Full	0.5	0.7	0.35	0.81	0.56	0.47
CLSI	enrofloxacin	Full	0.75	0.9	0.95	0.6	0.88	0.92
CLSI	gentamicin	Full	0.91	0.97	0.98	0.83	0.96	0.97
CLSI	marbofloxacin	Full	0.78	0.94	0.96	0.7	0.92	0.95
CLSI	orbifloxacin	Full	0.9	0.92	0.98	0.69	0.92	0.95
CLSI	TZP	Full	1	0.82	1	0.13	0.82	0.9
CLSI	pradofloxacin	Full	0.89	0.92	0.98	0.67	0.92	0.95
CLSI	ceftiofur	Full	0.82	0.87	0.93	0.69	0.85	0.9
ECOFF	ampicillin	Full	0.74	1	0.77	1	0.86	0.87
ECOFF	cefazolin	Full	0.67	0.77	0.92	0.36	0.75	0.84
ECOFF	cefpodoxime	Full	0.75	0.9	0.97	0.5	0.89	0.93
ECOFF	ceftazidime	Full	0.9	0.96	0.96	0.9	0.94	0.96
ECOFF	chloramphenicol	Full	0.91	0.91	0.99	0.56	0.91	0.95
ECOFF	doxycycline	Full	0.86	0.95	0.97	0.8	0.93	0.96
ECOFF	enrofloxacin	Full	0.53	0.96	0.84	0.83	0.84	0.9
ECOFF	gentamicin	Full	1	0.95	1	0.82	0.96	0.97
ECOFF	tetracycline	Full	0.94	0.96	0.97	0.93	0.96	0.97
ECOFF	TMP/SMX	Full	0.9	0.99	0.96	0.97	0.97	0.98
ECOFF	amoxicillin	Full	0.92	0.95	0.95	0.92	0.94	0.95
ECOFF	ceftiofur	Full	0.67	0.88	0.92	0.56	0.84	0.9
ECOFF	neomycin	Full	0.73	0.94	0.9	0.83	0.88	0.92
ECOFF	spectinomycin	Full	1	0.87	1	0.69	0.9	0.93
ECOFF	streptomycin	Full	0.89	0.92	0.96	0.8	0.91	0.94
ECOFF	co-ticarclav	Full	0.6	0.92	0.92	0.6	0.87	0.92
CLSI	amikacin	Count	1	0.93	1	0.29	0.93	0.96
CLSI	co-amoxiclav	Count	0.72	0.71	0.54	0.85	0.72	0.61
CLSI	ampicillin	Count	0.87	0.92	0.42	0.99	0.87	0.58
CLSI	cefazolin	Count	0.73	0.88	0.79	0.84	0.81	0.83
CLSI	cefovecin	Count	1	0.86	1	0.67	0.89	0.92
CLSI	cefpodoxime	Count	0.92	0.89	0.96	0.79	0.9	0.93
CLSI	ceftazidime	Count	1	0.89	1	0.78	0.92	0.94
CLSI	cephalexin	Count	0.77	1	0.63	1	0.83	0.77
CLSI	enrofloxacin	Count	1	1	1	1	1	1
CLSI	gentamicin	Count	0.91	1	0.98	1	0.99	0.99
CLSI	marbofloxacin	Count	1	1	1	1	1	1
CLSI	orbifloxacin	Count	1	1	1	1	1	1
CLSI	TZP	Count	1	0.87	1	0.17	0.87	0.93
CLSI	pradofloxacin	Count	1	0.98	1	0.9	0.98	0.99
CLSI	ceftiofur	Count	0.91	0.77	0.96	0.59	0.8	0.85
ECOFF	ampicillin	Count	0.79	1	0.81	1	0.89	0.89
ECOFF	cefazolin	Count	0.67	0.8	0.92	0.4	0.78	0.86
ECOFF	cefpodoxime	Count	0.75	0.94	0.97	0.6	0.91	0.95
ECOFF	ceftazidime	Count	0.9	0.88	0.96	0.75	0.89	0.92
ECOFF	chloramphenicol	Count	1	0.94	1	0.65	0.94	0.97
ECOFF	doxycycline	Count	0.86	0.98	0.97	0.92	0.96	0.98
ECOFF	enrofloxacin	Count	1	0.98	1	0.95	0.99	0.99
ECOFF	gentamicin	Count	1	0.96	1	0.85	0.97	0.98
ECOFF	tetracycline	Count	0.98	0.98	0.99	0.96	0.98	0.99
ECOFF	TMP/SMX	Count	1	1	1	1	1	1
ECOFF	amoxicillin	Count	1	0.95	1	0.92	0.97	0.98
ECOFF	ceftiofur	Count	0.8	0.85	0.95	0.55	0.84	0.9
ECOFF	neomycin	Count	1	1	1	1	1	1
ECOFF	spectinomycin	Count	1	0.87	1	0.69	0.9	0.93
ECOFF	streptomycin	Count	0.89	0.96	0.96	0.89	0.94	0.96
ECOFF	co-ticarclav	Count	0.6	0.92	0.92	0.6	0.87	0.92

CLSI	amikacin	Binary	1	0.6	1	0.069	0.61	0.75
CLSI	co-amoxiclav	Binary	1	0.14	1	0.72	0.74	0.25
CLSI	ampicillin	Binary	0.79	0.92	0.31	0.99	0.8	0.47
CLSI	cefazolin	Binary	0.23	0.96	0.59	0.83	0.62	0.73
CLSI	cefovecin	Binary	0.5	0.52	0.79	0.23	0.52	0.63
CLSI	cefpodoxime	Binary	1	0.29	1	0.38	0.5	0.44
CLSI	ceftazidime	Binary	1	0.37	1	0.37	0.54	0.54
CLSI	cephalexin	Binary	1	0.4	1	0.81	0.83	0.57
CLSI	enrofloxacin	Binary	0.88	0.59	0.96	0.29	0.63	0.73
CLSI	gentamicin	Binary	0.82	1	0.97	1	0.97	0.98
CLSI	marbofloxacin	Binary	1	0.31	1	0.2	0.41	0.47
CLSI	orbifloxacin	Binary	1	0.46	1	0.27	0.55	0.63
CLSI	TZP	Binary	1	0.18	1	0.031	0.21	0.31
CLSI	pradofloxacin	Binary	0.89	0.33	0.94	0.19	0.41	0.49
CLSI	ceftiofur	Binary	1	0.13	1	0.3	0.37	0.24
ECOFF	ampicillin	Binary	0.58	0.71	0.6	0.69	0.64	0.65
ECOFF	cefazolin	Binary	1	0.27	1	0.21	0.39	0.42
ECOFF	cefpodoxime	Binary	1	0.48	1	0.2	0.54	0.65
ECOFF	ceftazidime	Binary	0.6	1	0.87	1	0.89	0.93
ECOFF	chloramphenicol	Binary	0.91	1	0.99	1	0.99	0.99
ECOFF	doxycycline	Binary	0.86	0.98	0.97	0.92	0.96	0.98
ECOFF	enrofloxacin	Binary	0.16	1	0.76	1	0.77	0.86
ECOFF	gentamicin	Binary	0.96	0.99	0.99	0.96	0.98	0.99
ECOFF	tetracycline	Binary	0.98	0.98	0.99	0.96	0.98	0.99
ECOFF	TMP/SMX	Binary	0.95	1	0.98	1	0.99	0.99
ECOFF	amoxicillin	Binary	1	0.5	1	0.52	0.68	0.67
ECOFF	ceftiofur	Binary	0.8	0.66	0.94	0.34	0.69	0.78
ECOFF	neomycin	Binary	0.81	1	0.93	1	0.95	0.96
ECOFF	spectinomycin	Binary	1	0.87	1	0.69	0.9	0.93
ECOFF	streptomycin	Binary	0.89	0.96	0.96	0.89	0.94	0.96
ECOFF	co-ticarclav	Binary	1	0.32	1	0.23	0.43	0.48
